# Lupus-like nephritis with positive anti-neutrophil cytoplasmic antibodies and negative antinuclear antibodies

**DOI:** 10.1590/2175-8239-JBN-2020-0114

**Published:** 2020-10-26

**Authors:** Joana Eugénio Santos, Rita Vicente, Beatriz Malvar, Iolanda Santos, Miguel Coimbra, Manuel Amoedo, Carlos Pires

**Affiliations:** 1 Hospital Espírito Santo de Évora, Department of Nephrology, Évora, Portugal.

**Keywords:** Antineutrophil Cytoplasmic Antibodies, Antinuclear Autoantibodies, Lupus Nephritis, Lupus Erythematosus, Systemic, Glomerulonephritis, Anticorpos Anticitoplasma de Neutrófilos, Anticorpo Antinuclear, Nefrite Lúpica, Lúpus Eritematoso Sistêmico, Glomerulonefrite

## Abstract

Antineutrophil cytoplasmic antibodies (ANCAs) are associated with small vessel vasculitis but their prevalence is not rare in other immune diseases. In lupus nephritis (LN), their pathological role and clinical relevance have been the target of controversial views.

We present a case of acute kidney injury and nephrotic syndrome in a young woman with diffuse global proliferative and membranous nephritis on her kidney biopsy, showing a full-house immunofluorescence pattern, very allusive of class IV + V LN, but lacking associated clinical criteria and laboratory findings to support the diagnosis of systemic lupus erythematosus (SLE). Furthermore, the patient presented with high titers of ANCA, steadily decreasing alongside the renal function and proteinuria improvements, with mycophenolate mofetil (MMF) and steroid treatment.

The authors believe this is a case of lupus-like nephritis, in which ANCAs are immunological markers, although they are not directly involved in the pathogenesis.

## Resumo

Os anticorpos anticitoplasma de neutrófilos (ANCAs) estão associados à vasculite de pequenos vasos, no entanto, a sua prevalência não é rara em outras doenças imunológicas. Na nefrite lúpica (LN), o seu papel patológico e relevância clínica têm sido alvo de pontos de vista controversos.

Apresentamos um caso de lesão renal aguda e síndrome nefrótica em uma jovem com nefrite proliferativa difusa e membranosa em sua biópsia renal, muito alusivo a NL classe IV + V, com um padrão *full house* na imunofluorescência, mas sem critérios clínicos e achados laboratoriais para corroborar o diagnóstico de lúpus eritematoso sistêmico (LES). Não obstante, a paciente apresentou títulos elevados de ANCA, que diminuiram progressivamente com a melhoria da função renal e da proteinúria, após tratamento com micofenolato de mofetil (MMF) e esteróide.

Os autores acreditam que se trata de um caso de nefrite semelhante à nefrite lúpica, em que os ANCAs são marcadores imunológicos, embora não estejam diretamente envolvidos na patogênese.

## Introduction

Lupus nephritis (LN) is a complex immune glomerulonephritis that often complicates the clinical course of systemic lupus erythematosus (SLE). It occurs in 40% of SLE patients and is one of the most severe manifestations of the disease. It is a well-defined histological disease, characterized by deposition of immune complexes, at mesangial, subendothelial, and subepithelial locations of the glomeruli, and also extraglomerular alterations. Besides, these deposits may stain dominantly for IgG, and contain co-deposits of immunoglobulin A, immunoglobulin M, C3, and C1q, yielding the so-called "full house" immunofluorescence pattern, which is highly suggestive of LN.

There are cases of characteristic LN lacking clinical criteria for SLE, complicating the diagnosis. Such patients with microscopic and immunofluorescence findings, highly favoring the diagnosis of LN but lacking further signs, symptoms, or serologies, which confirm the diagnosis of SLE, may present distinct nephropathy. Other authors use two different terminologies concerning the disease: "lupus-like nephritis" and "idiopathic non-lupus full-house nephropathy"^2^^-^ 4.

The purpose of this article is to present a diagnosis of lupus-like nephritis, whose serological markers appear to be antineutrophil cytoplasmic antibodies (ANCA) instead of antinuclear autoantibodies (ANA), although they did not play a clear pathogenic role.

## Case Presentation

A 31-year-old Caucasian female presented with worsening lower limb and periorbital edema and fatigue. She had undergone routine blood and urine tests 4 months earlier, which had shown acute kidney injury, with a serum creatinine (sCr) of 1.8 mg/dL and new-onset microscopic hematuria.

Macroscopic urine abnormalities, oliguria, and lower urinary tract symptoms were absent. The patient also lacked skin lesions, joint pain or swelling, photosensitivity, oral ulcers, dyspnea, chest pain, fever, and constitutional symptoms. She was overweight (BMI 33.8 kg/m^2^ and had active smoking habits. Family history was negative for renal, autoimmune, or neoplastic diseases.

Physical examination was unimpressive other than high blood pressure and lower extremity 3+ pitting edema, extending from the mid-thigh to the feet.

Laboratory evaluation revealed kidney function deterioration (sCr 3.1 mg/dL and urea 100 mg/dL), normocytic and normochromic anemia (hemoglobin 9.4 g/dL), hypoalbuminemia of 1.5 g/dL and hyperlipidemia (total cholesterol 236 mg/dL). The remaining hematological series were unaffected. Her urinalyses presented significant proteinuria (>1000mg/dL) and >20 red blood cells per high power field, >50% dysmorphic. Heavy proteinuria was confirmed (19 g of protein excretion) in a 24-hour urine collection. On renal ultrasonography, kidneys had normal size and structure, with only a slight increase in parenchymal echogenicity.

Further autoimmune workup revealed low complement titers (45 mg/dL for C3 and 6 mg/dL for C4 fractions), and a positive ANCA test, with a very high titer (319 UI/L). She was negative for ANA and, consequently extractable nuclear antigen panel, antiphospholipid and lupus anticoagulant antibodies, anti-dsDNA, AMA, and anti-LLM. The ANCA found had a perinuclear pattern in immunofluorescence and reactivity against myeloperoxidase antigens (MPO-ANCA) in enzyme-linked immunosorbent assay. Human immunodeficiency virus and hepatitis screening tests showed no previous or current infections.

**Figure 1:**
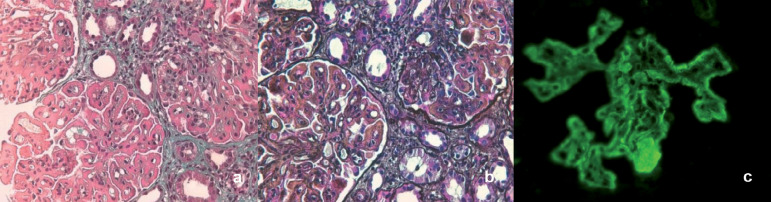
Image a (trichrome stain, x400) and b (silver methenamine stain, x400) shows endocapillary hypercellularity and prominent subendothelial deposits ("wire loops"). It shows a lobular expansion and increases of mesangial matrix, imparting a membranoproliferative glomerulonephritis-like appearance. We can see also global and diffuse thickening of the glomerular basal membrane with granular subepithelial deposits (mainly in trichrome) with silver-positive spikes. Tubules and interstitium present with extended mononuclear infiltrate and 60% of tubulointerstitial fibrosis. Image c (immunofluorescence, x400) shows IgG parietal granular deposits in glomeruli accompanied by IgA, IgM, C3, C1q, kappa, and lambda deposits in a full-house pattern.

Kidney biopsy (Figure 1) was performed, revealing active global endocapillary and mesangial hypercellularity, and a diffuse thickening of the glomerular wall with "spikes" on light microscopy. On immunofluorescence there were diffuse mesangial, subendothelial, and subepithelial immune deposits, in "full-house nephropathy" pattern. The pathological diagnosis was consistent with class IVa and V LN.

Intravenous methylprednisolone was prescribed in pulses for 3 consecutive days, followed by 1 mg/kg oral prednisolone and 3 g MMF per day. By the second month of treatment, there was a modest improvement in proteinuria and kidney function (24-h urine protein excretion and sCr decreased to 13g and 1.8 mg/dL respectively) and there were fewer dysmorphic red blood cells in the urinary sediment. The immune response was also positive: MPO-ANCA titers fell to 33 IU/L and serum complement levels progressively increased.

The patient's recovery was remarkable in the following 4 months. On the 6^(th)^ month of immunosuppressive (IS) therapy (after prednisolone slow tapering in the previous months to 5 mg/day), there was a complete remission with sCr 1.0 g/dL, proteinuria 0.6 g/24 hour-urine, resolution of microscopic hematuria, and normalization of serum complement levels. The titers of MPO-ANCA steadily declined and MMF was tapered to a maintenance dose of 500 mg twice daily. SLE associated antibodies, ANA, and dsDNA remained negative.

After 2 years of a complete sustained response, with a daily urine protein excretion of 300 mg, no hematuria, and normal kidney function, ANCA titers became negative. The patient then switched to azathioprine, motivated by her wishes to conceive. The patient has now 7 years of follow-up on remission of her nephritis. Throughout all of the follow-up period, there have never been signs of systemic vasculitis, and the patient has never experienced extrarenal manifestations of SLE or nephritis flare.

## Discussion

ANAs are thought to play an essential role in the pathogenesis of LN but its pathogenesis remains incompletely understood. The theory describing that immune complexes in circulation become passively trapped in glomeruli is not supported by current evidence and is currently considered unlikely^5^.

The recent discovery of a familiar form of SLE, linked to a mutation of the gene encoding DNase 1L3^6^, and Sisirak et al.'s report of an experimental nephritis with autoimmunity to DNA caused by DNase 1L3 deficience DNase 1L3^7^ have altogether proved that gene deficiencies in chromatin metabolism may result in a lupus-like phenotype. Chromatin fragments are released from dying renal cells, not appropriately degraded during the programmed cell death process because of the acquired loss of the renal endonuclease DNasel. These fragments are then targeted by anti-chromatin (anti-nucleosome) antibodies, including anti-dsDNA. Also, the accumulation of neutrophil extracellular traps (NETs) deposits in the glomerular capillaries, prompting tissue injury and further cell death^8^.

Some animal models of LN demonstrated that autoreactive T cells-induced damage accounted for a significant part of the kidney injury^9^, proving that cross-reaction of anti-dsDNA antibodies with intrinsic renal antigens is not essential for the development of LN^10^. Therefore, the absence of circulating anti-chromatin antibodies is not a reason to rule out this diagnosis.

Patients with active LN may have other autoantibodies, including ANCA^11^. ANCA's presence in LN patients has been the target of controversial views concerning its clinical relevance and role^12^. Retrospective studies found a high prevalence of ANCA in LN^13^ and a positive correlation with disease activity and severity. A recent report showed that ANCAs were associated with more endocapillary hypercellularity, necrosis, crescent formation, interstitial inflammation, tubular atrophy, and interstitial fibrosis^14^^,^ 15. On the other hand, MPO-ANCA were more likely to have the last two characteristics, and lower titers of ANA or anti-dsDNA at the time of biopsy^16^.

Independently of ANCA presence, features of vasculitis such as hyaline and non-inflammatory necrotizing lesions, lymphocytic infiltration of the vessel wall, and more rarely arteriolar thrombi may all be found in LN^1^^)(^ 7. In our patient's nephritis, in spite of apparent involvement of MPO-ANCA, there were no characteristic necrotizing lesions on her biopsy. ANCAs have been linked to conditions with increased turnover and impaired non-apoptotic clearance of neutrophils in probable relation with NETs, which act as prominent autoantigens, leading to disease-relevant autoantibody production^18^ not leading to the development of significant histological findings.

We were faced with biopsy-proven lupus-like nephritis, with circulating ANCA, without SLE diagnostic criteria. Although the diagnosis of SLE cannot be established solely based upon renal biopsy findings, some pathological findings of LN often exclude other diagnoses. That is the case of overlapping glomerular membranous and diffuse proliferative lesions, and the so-called "full-house" immunostaining, both present in the kidney biopsy of this patient.

This pathological pattern is not specific for LN. In a young person, after dropping HCV and HIV immune complex-mediated glomerulonephritis, infection-related glomerulonephritis and fibrillary glomerulonephritis, a diagnosis of lupus-like nephritis is more likely. In our patient, the time of disease outbreak, the absence of dysproteinemia, malignancy and other auto-immune diseases, as well as the remission of the disease with MMF and corticosteroids, made the fibrillary glomerulonephritis diagnosis unlikely, once it is characterized by a poor prognosis, with a progress to end-stage renal disease within few years^19^.

In this patient, not only was circulating ANCA detected but also its levels correlated with disease activity: extremely positive at the time of diagnosis, progressively declining with disease improvement and becoming negative at clinical remission. It did not have a clear pathogenic role, and its frequency can be explained by NETosis, which provide a source of autoantigens.

The renal-limited nature of the patient's disease, without further signs or symptoms of SLE, and negative anti-chromatin autoantibodies, makes the diagnosis of SLE unviable. However, the negative serology does not exclude LN, as it can be explained by autoreactive T cells, ANA's entrapped in circulating immune complexes, low levels of antibodies or their loss through the kidney in patients with severe proteinuria^20^.

In conclusion, the authors believe that it is a case of lupus-like nephritis with circulating ANCA and good prognosis. After careful etiological investigation and under favorable and sustained clinical response to MMF and steroids, lupus-like nephritis is the most likely situation, in possible relation with SLE. Once antinuclear antibodies, symptoms, and signs of the disease become detectable after several years of follow-up, the authors suggest patients' monitoring for the development of SLE.
